# Comparison of delayed versus immediate coloanal anastomosis in patients with low rectal cancer: a recent meta-analysis

**DOI:** 10.3389/fonc.2025.1749894

**Published:** 2026-01-26

**Authors:** Yonghong Wang, Ke Liu, Wenjie Zhou, Jie Dan, Mingjie Zhu, Lei Chen, Wanbin He, Ming Li, Jiangpeng Li

**Affiliations:** Department of Gastrointestinal Surgery, The People’s Hospital of Leshan, Leshan, Sichuan, China

**Keywords:** complications, delayed coloanal anastomosis, immediate coloanal anastomosis, low rectal cancer, meta-analysis

## Abstract

**Background:**

In sphincter-preserving surgery for low rectal cancer (LRC), Immediate coloanal anastomosis (ICA) combined with prophylactic ileostomy remains the standard approach. However, this procedure is associated with the need for a second operation to reverse the stoma and risks of stoma-related complications. Delayed coloanal anastomosis (DCA) has recently regained attention as an alternative strategy, particularly in the context of evolving principles of minimally invasive surgery and enhanced recovery after surgery (ERAS). This meta-analysis aimed to systematically evaluate and compare DCA and ICA in terms of perioperative outcomes, postoperative complications, and oncological efficacy.

**Methods:**

Following the PRISMA guidelines, we conducted a comprehensive literature search across PubMed, MEDLINE, Embase, Cochrane Library, Web of Science, and the Chinese Biomedical Literature Database (CBM) from database inception to October 2025. We included clinical studies comparing DCA and ICA for the treatment of LRC. The Risk of Bias 2 (ROB2) tool was used to assess bias in randomized controlled trials (RCTs), and the ROBINS-I tool was applied for non-randomized studies. Meta-analyses were performed using R 4.2.0 and RevMan 5.3 software.

**Results:**

A total of 16 studies (2 RCTs and 14 retrospective cohort studies) involving 1,409 patients (822 in the ICA group and 587 in the DCA group) were included. No statistically significant differences were observed between the two groups in operative time (SMD = 0.10, 95% CI: -0.23 to 0.44, P = 0.55), intraoperative blood loss (SMD = 0.34, 95% CI: -0.19 to 0.86, P = 0.21), or length of hospital stay (SMD = -0.37, 95% CI: -1.14 to 0.40, P = 0.34). However, the ICA group had significantly higher risks of total complications (OR = 2.74, 95% CI: 1.89–3.98, P < 0.00001), anastomosis-related complications (OR = 3.46, 95% CI: 2.32–5.15, P < 0.00001), and postoperative anastomotic leakage (OR = 2.79, 95% CI: 1.71–4.57, P < 0.0001) compared to the DCA group. There were no significant differences in local recurrence rate (OR = 1.02, 95% CI: 0.40–2.63, P = 0.98) or distant metastasis rate (OR = 1.51, 95% CI: 0.89–2.54, P = 0.13). Publication bias assessment revealed no substantial asymmetry in key outcomes, and sensitivity analyses confirmed the stability and robustness of the findings.

**Conclusion:**

Compared with ICA, DCA is associated with significantly lower risks of overall complications, anastomotic complications, and anastomotic leakage in sphincter-preserving surgery for LRC, without compromising oncological safety. It demonstrates comparable performance in core perioperative indicators and may offer particular advantages for patients seeking minimally invasive approaches, those unable to tolerate stomas, or those at high risk of anastomotic failure. Therefore, DCA represents a viable and potentially preferable surgical option in the management of LRC. This study strictly adhered to the Preferred Reporting Items for Systematic Reviews and Meta-Analyses (PRISMA) statement and was registered on the PROSPERO international systematic review registration platform (registration number: CRD420251233006).

## Introduction

Low rectal cancer (LRC) is defined as a malignant tumor whose distal margin lies ≤5 cm from the anal verge, representing 30% to 40% of all rectal cancers globally (1). In recent years, with the widespread adoption of sphincter-preserving surgical techniques, an increasing number of scholars have proposed extending the anatomical boundary to include tumors located within 7 cm of the anal verge under the category of LRC (2). The central challenge in surgical management lies in achieving a balance between oncological radicality and preservation of anal sphincter function. Consequently, sphincter-preserving surgery has become the preferred approach for patients who meet anatomical and functional criteria (3). Currently, the standard strategy to mitigate anastomotic complications in LRC surgery is immediate coloanal anastomosis (ICA) combined with prophylactic terminal ileostomy. This involves performing a low anastomosis followed by creation of a protective stoma. Approximately 3 to 4 months postoperatively, if imaging and clinical assessments confirm adequate healing, a second procedure—ileostomy reversal—is performed. While this two-stage approach effectively reduces the clinical impact of anastomotic leakage, it introduces additional burdens, including the necessity of a second operation, challenges in stoma care, and risks of stoma-related complications. In recent years, the advancement of minimally invasive techniques—particularly natural orifice specimen extraction surgery (NOSES)—has promoted the concept of “scarless” abdominal surgery, gaining increasing acceptance in colorectal oncology (3, 4). In selected patients, NOSES enables specimen removal through the anus, eliminating the need for an abdominal incision and thereby enhancing cosmetic and functional outcomes. However, the placement of a prophylactic ileostomy compromises this minimally invasive advantage by requiring a separate abdominal opening, thus undermining one of the core principles of NOSES.

In contrast, delayed coloanal anastomosis (DCA), exemplified by the Turnbull-Cutait procedure, was first described independently by Turnbull and Cutait in 1961. Initially applied for benign conditions such as Hirschsprung’s disease and Chagas-related megacolon, as well as for rectal cancer, DCA has recently regained interest in the context of evolving paradigms in minimally invasive surgery (MIS) and enhanced recovery after surgery (ERAS) (5, 6). This technique follows a two-stage protocol: during the first stage, an abdominoperineal pull-through resection is performed, with the proximal colon exteriorized through the anus and secured externally. Five to ten days later, the second stage involves excising the exteriorized bowel segment and completing the coloanal anastomosis. Notably, since no anastomosis is created in the initial phase, the risk of early anastomotic complications is eliminated. Any potential complications related to the final anastomosis occur only after the second procedure and are generally less severe. Furthermore, because the entire procedure avoids an abdominal stoma and can be integrated with transanal specimen extraction, it aligns closely with the principles of NOSES (7). Nevertheless, the transanal exteriorization of the colon presents unique technical challenges and requires specialized nursing care, potentially increasing perioperative morbidity.

Although previous systematic reviews and meta-analyses have compared these two anastomotic strategies, existing evidence remains limited by several key issues. Most included studies originate from Western centers, limiting generalizability to other populations. Additionally, many reports feature short follow-up durations, resulting in insufficient data on long-term oncological outcomes such as local recurrence and distant metastasis. Some analyses have also inadvertently included patients with mid- or high rectal cancer, introducing heterogeneity and potentially biasing results. To address these limitations, this updated meta-analysis incorporates the most recently published comparative studies, focusing exclusively on patients undergoing sphincter-preserving surgery for LRC, to provide a more accurate and comprehensive evaluation of the perioperative and oncological differences between ICA and DCA.

## Methods

### Literature search strategy

This study strictly adhered to the Preferred Reporting Items for Systematic Reviews and Meta-Analyses (PRISMA) statement and was registered on the PROSPERO international systematic review registration platform (registration number: CRD420251233006). We conducted a systematic search of the following databases: PubMed, MEDLINE, Embase, Cochrane Library, Web of Science, and China Biomedical Literature Database (CBM). The search period covered the establishment of each database to October 30, 2025. The search strategy was developed based on the PICOS framework (**Supplementary Table 1**), combining MeSH terms and free words to form search terms for key concepts such as low rectal cancer, anus-preserving surgery, delayed anastomosis, and primary anastomosis. The specific search strategies for each database are detailed in **Supplementary Table 2** of the supplementary materials. To minimize omissions, we also used backward citation tracking to manually screen the references of included studies. The literature screening process was independently completed by two researchers using a pre-designed standardized form for data extraction. Any discrepancies were resolved through discussion with a third senior researcher.

### Inclusion and exclusion criteria

The inclusion criteria were as follows: study types were randomized controlled trials (RCTs), cohort studies, or case-control studies; the study subjects were clearly defined as patients with LRC, with the lower edge of the tumor ≤ 7 cm from the anal verge; the intervention measures were delayed anastomosis and primary anastomosis; the outcome measures included at least one of the following: operation time, intraoperative blood loss, overall postoperative complication rate, anastomotic-related complication rate (such as anastomotic leakage, anastomotic stenosis, etc.); language was limited to Chinese and English; there was no time limit on publication to obtain as much research data as possible.

The exclusion criteria were: non-comparative studies, such as those reporting only the effect of a single surgical method; studies with subjects who were not LRC patients or had other conditions that significantly affected the efficacy and safety of the surgery; studies with incomplete data that could not be supplemented by contacting the authors; duplicate publications, with the version having more complete and higher-quality data retained.

### Data extraction

Two researchers independently extracted key information from the included studies based on pre-defined criteria. The extracted information included the first author, publication year, study type, sample size (number of cases in the delayed anastomosis group and the primary anastomosis group), patient characteristics (age, gender, BMI, distance from the tumor to the anal verge, etc.), perioperative outcomes (operation time, intraoperative blood loss, hospital stay), postoperative complications (total complications, anastomotic leakage, anastomotic stenosis, anastomotic bleeding, pelvic infection, postoperative intestinal obstruction, urinary difficulty), and tumor outcomes (local recurrence, distant metastasis). When relevant data were not clearly stated, we attempted to contact the authors for supplementation. Discrepancies in data extraction were resolved through discussion with a third reviewer.

### Quality assessment of literature

Risk of bias assessment: The risk of bias for randomized trials was evaluated by two reviewers using the Cochrane Risk of Bias Tool version 2 (ROB2). Each study was assessed in five aspects, with the results classified as “low risk”, “some concerns”, or “high risk”. For non-randomized controlled trials, the Risk of Bias in Non-randomized Studies - of Interventions (ROBINS-I) tool was used to evaluate from seven domains. Each domain was judged as “low risk”, “moderate risk”, “high risk”, or “serious risk”. Any disagreements were resolved through discussion involving a third reviewer.

### Statistical analysis

Data analysis was conducted using R 4.2.0 and RevMan 5.3 statistical software. For binary variables, such as the overall postoperative complication rate and anastomotic-related complication rate, the odds ratio (OR) and its 95% confidence interval (CI) were used as the effect size; for continuous variables, such as operation time and intraoperative blood loss, the standardized mean difference (SMD) and its 95% CI were used as the effect size. The Cochran Q test was used to assess heterogeneity among studies, and the I² statistic was calculated (I² > 50% indicated significant heterogeneity). To fully consider the clinical and methodological heterogeneity among studies, a random-effects model was used for the meta-analysis. Sensitivity analysis was performed by sequentially excluding individual studies to observe the changes in the combined effect size and evaluate the stability of the results. Publication bias was detected using a funnel plot combined with Egger’s test. If the funnel plot was approximately symmetrical and the P value of Egger’s test was greater than 0.05, it indicated a small publication bias and high reliability of the results. All hypothesis tests were two-sided, and the statistical significance was set at P < 0.05.

## Results

### Literature search and characteristics of included studies

**Figure 1:**
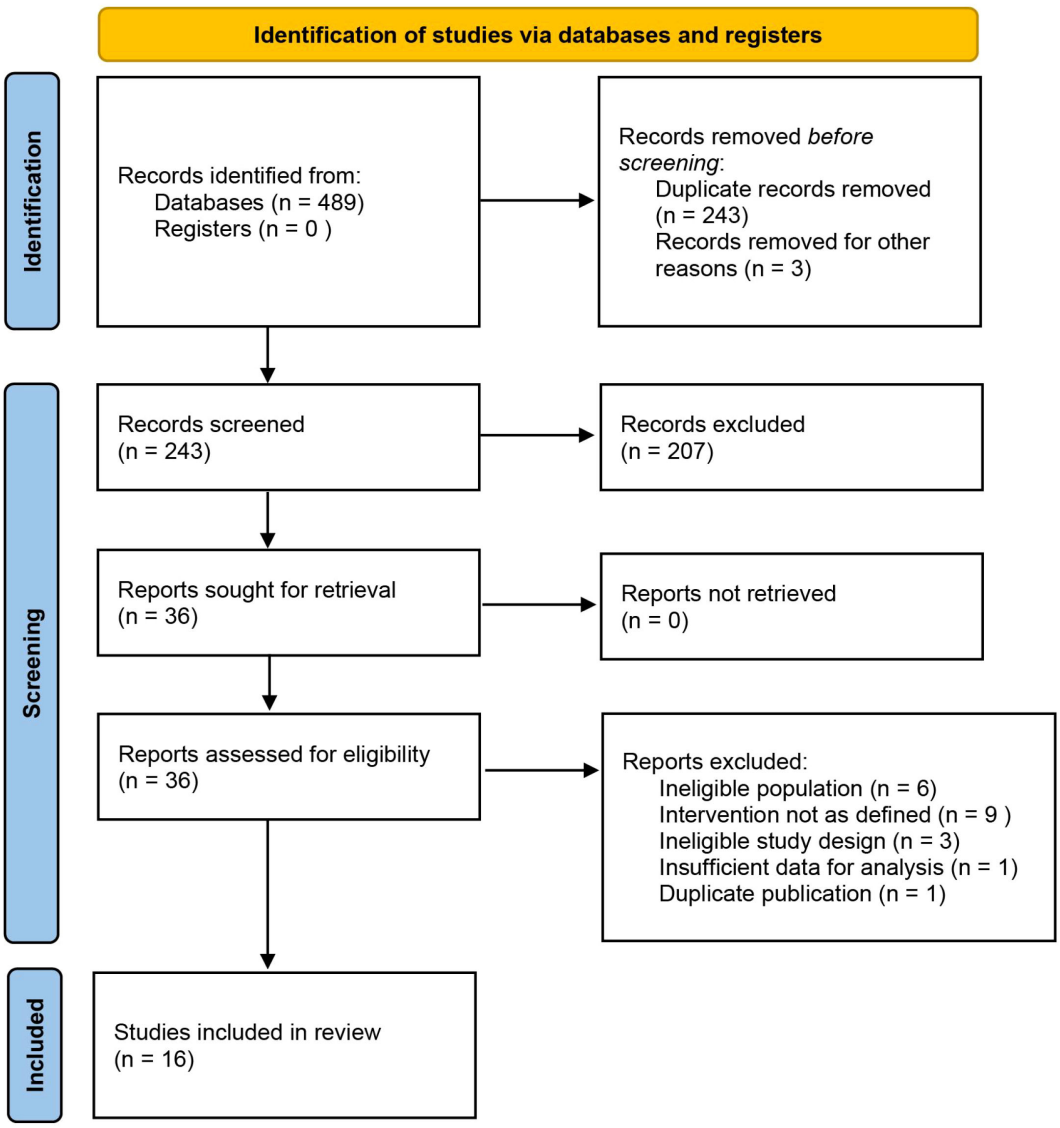
Flowchart of study selection and inclusion based on the PRISMA guidelines for reporting systematic reviews and meta-analyses.

A total of 489 records were identified through systematic database searching. After removing 243 duplicates and excluding 3 records that failed to meet preliminary screening criteria, 243 articles proceeded to full-text assessment. Of these, 20 were excluded for the following reasons: ineligible study population (n = 6), inconsistent interventions (n = 9), inadequate study design (n = 3), insufficient data reporting (n = 1), or duplicate publication (n = 1). Ultimately, 16 clinical studies met the inclusion criteria and were included in the meta-analysis (**Figure 1**). The included studies were published between 2016 and 2025 and comprised 2 RCTs (8, 9) and 14 retrospective cohort studies (10–23), including one international multicenter study (Daichi 2025). The total sample size was 1,409 patients, with 822 assigned to the ICA group and 587 to the DCA group. Baseline characteristics—including age, sex, body mass index (BMI), and tumor distance from the anal verge—were well balanced between the two groups, with no clinically significant differences, indicating adequate comparability across studies (**Table 1**). The detailed surgical approach types of the ICA and DCA groups included in the studies are presented in **Supplementary Table 3**.

**Table 1:** Baseline data of the patients in the included studies.

Author	Publication year	Country	Study type	Sample size (n)		Age (y)		Sex (M/F)		BMI		Distance from the anal verge (cm)	
				ICA	DCA	ICA	DCA	ICA	DCA	ICA	DCA	ICA	DCA
Xiong	2016	China	Retrospective	56	72	56.0 ± 11.5	59.0 ± 10.3	38/18	44/28	23.6 ± 3.4	24.3 ± 3.2	5.6 ± 0.8	5.5 ± 0.6
Zhou	2017	China	Retrospective	40	40	54 ± 8	55 ± 8	20/20	21/19	NA	NA	3-6	
Liu	2020	China	Retrospective	33	30	54.8 ± 5.1	55.2 ± 4.6	17/16	16/14	NA	NA	5.8 ± 0.9	5.7 ± 0.9
Luo	2020	China	Retrospective	30	32	57.3 ± 7.2	58.7 ± 7.7	20/10	21/11	22.8 ± 2.6	22.8 ± 3.4	3.7 ± 0.09	3.77 ± 0.09
Biondo	2020	Spain and Italy	RCT	46	46	63.8 ± 7.7	58.9 ± 10.1	37/9	35/11	26.0 ± 2.7	26.0 ± 1.63	5.0 (4.0-6.0)	5.0 (4.0-6.0)
Guo	2021	China	Retrospective	25	27	62.4 ± 7.6	58.8 ± 8.9	NA	NA	21.2 ± 3.4	24.6 ± 3.5	5.76 ± 1.13	4.39 ± 0.94
Tang	2021	China	Retrospective	33	26	51 ± 8	50 ± 7	16/17	12/14	22.7 ± 2.8	23.7 ± 1.9	2.56 ± 0.76	2.92 ± 0.77
Li	2021	China	Retrospective	35	30	64. 7 ± 1. 6	65.4 ± 2.0	NA	NA	25.1 ± 2.6	26.0 ± 1.4	2-6	
Guner	2021	Turkey	Retrospective	11	22	52.5 ± 8.2	57.1 ± 10.1	6/5	11/11	29.0 ± 2.0	29.8 ± 4.0	3.3 ± 0.6	4.0 ± 1.5
Madbouly	2022	Egypt	Retrospective	25	20	47.7 ± 12.3	51.6 ± 9.1	13/12	12/8	27 ± 2.7	26 ± 2.7	6 (5–7)	6 (5–7)
Majbar	2022	Morocco and France	Retrospective	26	19	63 ± 7	53 ± 3	15/11	12/7	24.6 ± 4.8	22.5 ± 4.0	4 ± 0.55	5 ± 0.25
Melka	2022	France	Retrospective	111	33	59 ± 11	60 ± 12	85/26	23/10	25 ± 4	24 ± 5		
Fu	2022	China	Retrospective	44	30	65.7 ± 10.4	65.5 ± 11.4	28/16	13/17	23.3 ± 3.0	22.6 ± 2.6	<5	
Zhou	2022	China	RCT	39	39	6.1 ± 4.3	56.2 ± 5.2	23/16	22/17	23.2 ± 2.0	23.2 ± 2.2	3.01 ± 0.33	3.02 ± 0.25
Seow-En	2024	Singapore	Retrospective	72	12	67.2 ± 9.9	69.3 ± 6.2	44/28	8/4	22.9 ± 2.9	23.0 ± 3.7	<6	
Daichi	2025	Japan	Retrospective*	196	109	61 ± 14.5	62 ± 11.5	147/49	82/27	23.6 ± 6.2	23.9 ± 5.7	4.0 ± 1.7	4.8 ± 1.7

### Meta-analysis results

#### Perioperative outcomes

**Figure 2:**
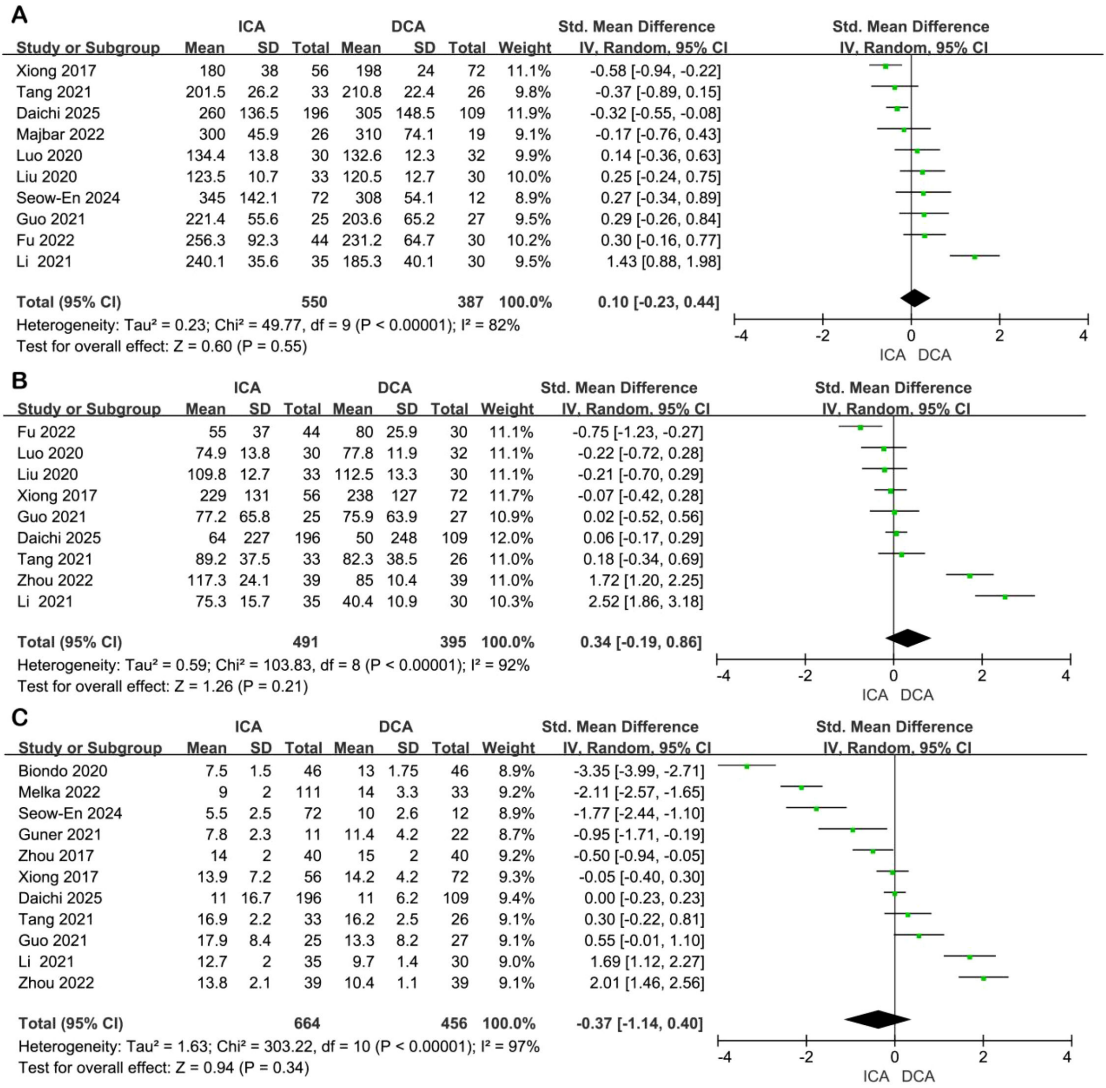
Forest plot of comparison of ICA versus DCA for perioperative outcomes. **(A)** Forest plot for operative time; **(B)** Forest plot for intraoperative blood loss; **(C)** Forest plot for length of stay in hospital.

**Operation time**: Data on operation time were reported in 10 studies. Significant heterogeneity was observed across studies (I² = 82%, P < 0.00001), prompting the use of a random-effects model. Pooled analysis revealed no statistically significant difference between the ICA and DCA groups (standardized mean difference [SMD] = 0.10, 95% confidence interval [CI]: −0.23 to 0.44, P = 0.55) (**Figure 2A**).

Intraoperative blood loss: Nine studies provided data on intraoperative blood loss. High heterogeneity was present (I² = 92%, P < 0.00001), and a random-effects model was applied. The combined estimate showed no significant difference between groups (SMD = 0.34, 95% CI: −0.19 to 0.86, P = 0.21) (**Figure 2B**).

Length of hospital stay: Eleven studies contributed data on postoperative hospitalization duration. Substantial heterogeneity was detected (I² = 97%, P < 0.00001), warranting the use of a random-effects model. Meta-analysis indicated no statistically significant difference in length of stay between the ICA and DCA groups (SMD = −0.37, 95% CI: −1.14 to 0.40, P = 0.34) (**Figure 2C**).

#### Postoperative complications

**Figure 3:**
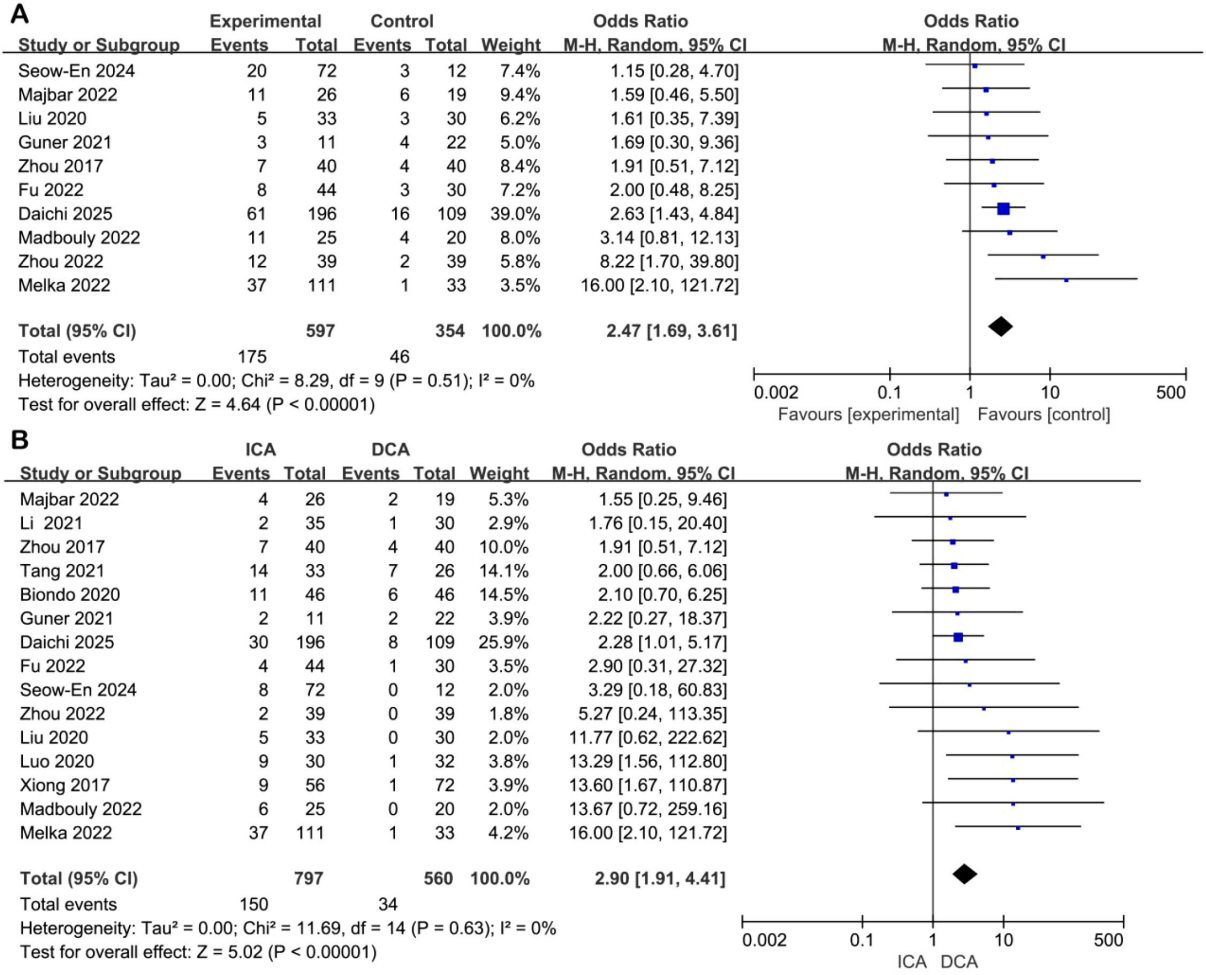
Forest plot of comparison of ICA versus DCA for postoperative complications. **(A)** Forest plot for overall complication; **(B)** Forest plot for anastomotic-related complications.

Total complications: Ten studies reported on overall postoperative complications. No heterogeneity was observed (I² = 0%), and a random-effects model was used due to anticipated clinical diversity. The ICA group had a significantly higher risk of total complications compared to the DCA group (odds ratio [OR] = 2.74, 95% CI: 1.89–3.98, P < 0.00001) (**Figure 3A**).

Anastomosis-related complications: Fifteen studies contributed data on anastomotic complications, including leakage, stricture, and bleeding. Homogeneity was high (I² = 0%), and the random-effects model was retained for methodological consistency. The pooled result demonstrated a significantly increased risk in the ICA group (OR = 3.46, 95% CI: 2.32–5.15, P < 0.00001) (**Figure 3B**).

**Figure 4:**
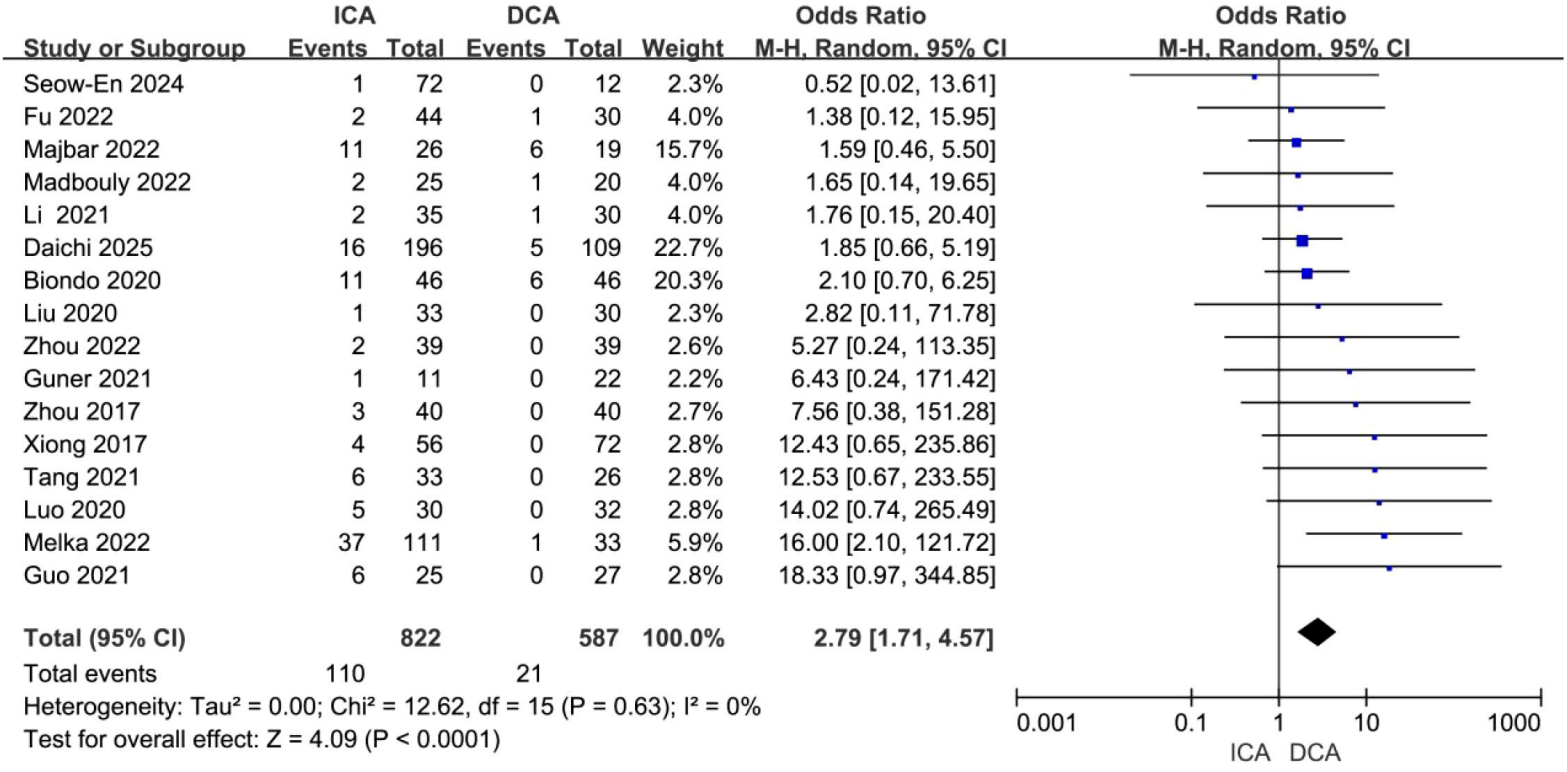
Forest plot of comparison of ICA versus DCA for anastomotic leak.

Postoperative anastomotic leakage: All 16 included studies reported anastomotic leakage outcomes. There was no statistical heterogeneity (I² = 0%), and the random-effects model was applied. Patients in the ICA group exhibited a significantly higher risk of postoperative anastomotic leakage than those in the DCA group (OR = 2.79, 95% CI: 1.71–4.57, P < 0.0001) (**Figure 4**).

Specific postoperative complications: Additional analyses of specific complications were conducted, with results summarized in **Table 2**. Nine studies reported anastomotic bleeding, and no statistically significant difference was found between the two groups (OR = 2.36, 95% CI: 0.83–6.75, P = 0.11). Anastomotic stricture data were available from 9 studies, showing no significant between-group difference (OR = 1.97, 95% CI: 0.89–4.37, P = 0.10). Seven studies reported pelvic infection, with the ICA group having a significantly higher risk (OR = 2.56, 95% CI: 1.29–5.10, P < 0.01). Intestinal obstruction was evaluated in 6 studies, and no significant difference was observed (OR = 2.40, 95% CI: 0.66–8.74, P = 0.19). Acute urinary retention was reported in 7 studies, with no statistically significant difference between groups (OR = 1.21, 95% CI: 0.49–3.01, P = 0.67).

**Table 2:** The results of the meta-analysis on specific postoperative complications.

Complication	Number	OR	95%CI	P-value
Anastomotic leak	16	2.79	1.71 – 4.57	<0.01
Anastomosis bleeding	9	2.36	0.83 – 6.75	0.11
Anastomotic stricture	9	1.97	0.89 – 4.37	0.10
Pelvic infection	7	2.56	1.29 – 5.10	<0.01
Intestinal obstruction	6	2.40	0.66 – 8.74	0.19
Acute urinary retention	7	1.21	0.49 – 3.01	0.67

#### Oncological outcomes

**Figure 5:**
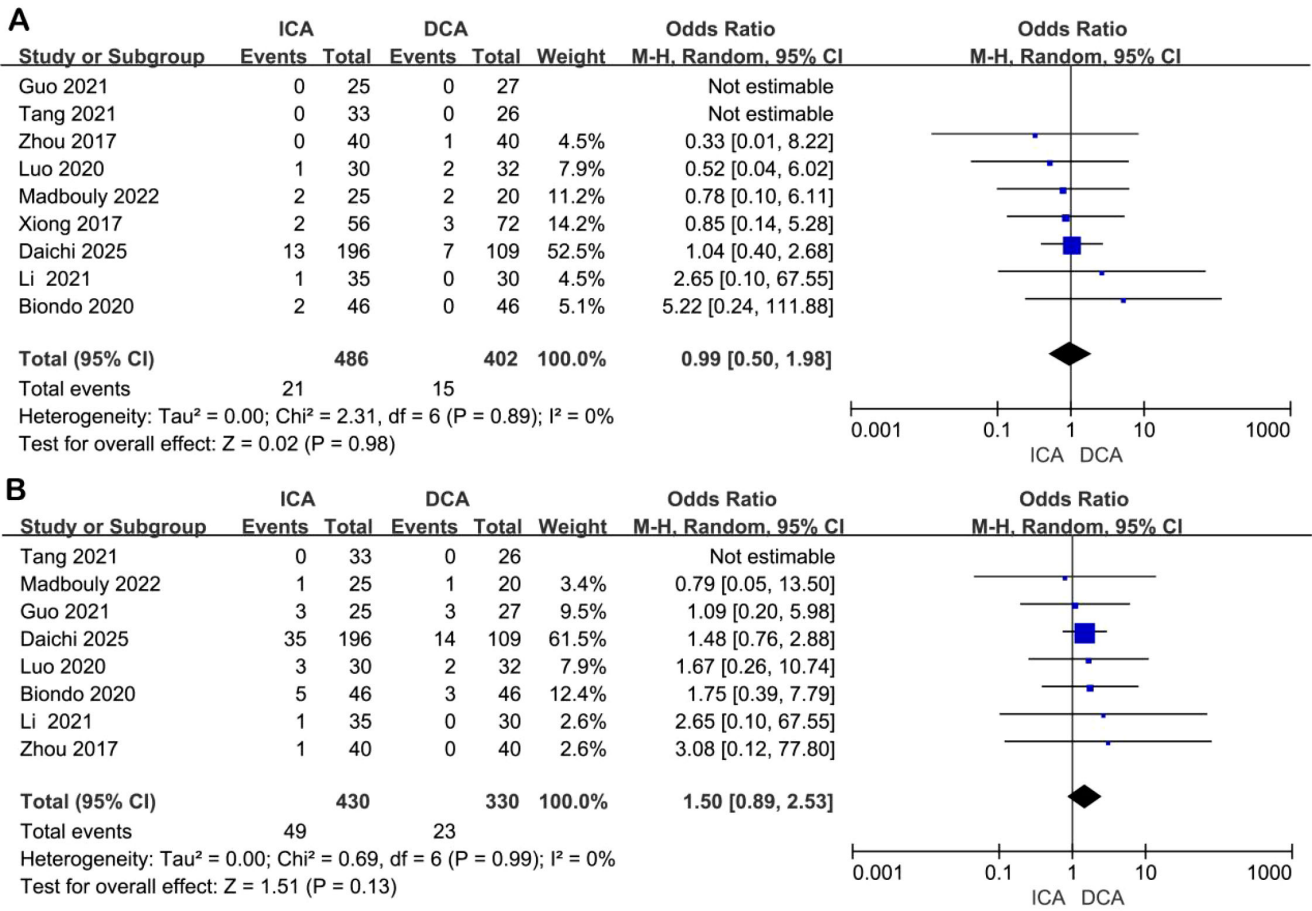
Forest plot of comparison of ICA versus DCA for oncologic outcomes. **(A)** Forest plot for local recurrence; **(B)** Forest plot for distant metastases.

Local recurrence: Nine studies provided data on local recurrence (effect size could not be calculated in two studies due to missing event counts). No heterogeneity was detected (I² = 0%), and a random-effects model was used. There was no statistically significant difference in local recurrence rates between the ICA and DCA groups (OR = 1.02, 95% CI: 0.40–2.63, P = 0.98) (**Figure 5A**).

Postoperative distant metastasis: Eight studies reported distant metastasis outcomes (one study excluded from pooling due to incomplete data). Heterogeneity was absent (I² = 0%), and the analysis was conducted using a random-effects model. No significant difference was observed in the rate of distant metastasis between the two groups (OR = 1.51, 95% CI: 0.89–2.54, P = 0.13) (**Figure 5B**).

#### Quality assessment of included studies

Risk of bias assessment was performed using appropriate tools based on the type of included studies, and the results indicated that the overall quality of the literature was good with controllable bias. For the 2 RCTs, the Cochrane Risk of Bias Tool version 2 (ROB2) was used to evaluate five core domains, including randomization process, deviations from intended interventions, and missing outcome data. All domains were classified as “low risk of bias”, with no evidence of irregular randomization, data missing, or selective reporting. This confirmed the high scientific rigor in the design and implementation of these RCTs (**Supplementary Figure 1**).

For the 14 retrospective cohort studies, the Risk of Bias in Non-randomized Studies of Interventions (ROBINS-I) tool was applied to assess seven domains, such as confounding factors, selection of participants, and exposure assessment. The results showed that 12 studies had an overall “moderate risk of bias”, while 2 studies (Luo 2020, Madbouly 2022) were rated as “low risk of bias”; no “high risk” or “serious risk” studies were identified (**Supplementary Table 4**). Among these domains, confounding bias and selection bias were mostly “moderate risk”, mainly due to insufficient adjustment for potential confounders in some studies or incomplete description of participant screening procedures. In contrast, all studies were rated “low risk” in domains of exposure assessment, misclassification during follow-up, and missing data, with low risks of outcome measurement bias and selective reporting bias, which did not significantly affect the reliability of the study results.

#### Sensitivity analysis and publication bias assessment

Sensitivity analysis was conducted by sequentially excluding each included study and recalculating the pooled effect size to assess the impact of individual studies on the overall results. Results showed that after excluding any single study, the pooled odds ratios (OR) for total complications, anastomosis-related complications, and anastomotic leakage fluctuated within a narrow range (total complications: OR 2.51-2.98; anastomotic leakage: OR 2.62-2.97), all maintaining statistical significance (all P < 0.001). No outlier studies that significantly affected the stability of the results were identified, confirming the robustness and reliability of the conclusions in this study.

Publication bias was evaluated using funnel plots combined with Egger’s test. The funnel plots for primary outcome indicators (including operative time, intraoperative blood loss, and total complication rate) were all roughly symmetrical, and Egger’s test results showed all P-values > 0.05, indicating no significant publication bias. Only the Egger’s test for length of hospital stay yielded P = 0.02, which was attributed to definition heterogeneity of “length of hospital stay” across studies (some only counted the first-stage surgery, while others included the two-stage surgery) (**Supplementary Figure 2**). This was not true publication bias and did not affect the reliability of the overall results.

## Discussion

The central challenge in sphincter-preserving surgery for LRC lies in achieving a balance between oncological radicality, functional preservation, and minimization of postoperative complications. ICA combined with prophylactic ileostomy remains the current standard approach, effectively mitigating the clinical consequences of anastomotic leakage. However, this strategy has obvious limitations, including the risk of stoma-related complications, the need for a second operation to close the stoma, and the negative impact on the quality of life of patients (10, 11). In contrast, the classic Turnbull-Cattet procedure, a representative example of DCA, has experienced a resurgence in the era of minimally invasive surgery and ERAS protocols. By deferring anastomosis to a second stage and avoiding prophylactic stomas, DCA offers a theoretically safer alternative (24, 25). Although previous meta-analyses have examined this comparison, the accumulating evidence—particularly long-term oncological outcomes and recent high-quality comparative studies—highlights the need for an updated comprehensive analysis. Therefore, this study presents a comprehensive systematic review and meta-analysis incorporating the latest clinical data to provide a more robust and current evidence base for evaluating the relative merits of ICA and DCA.

Despite DCA involving a two-stage surgical process, our analysis revealed no statistically significant differences between the DCA and ICA groups in operation time (SMD = 0.10, 95% CI: −0.23 to 0.44, P = 0.55), intraoperative blood loss (SMD = 0.34, 95% CI: −0.19 to 0.86, P = 0.21), or length of hospital stay (SMD = −0.37, 95% CI: −1.14 to 0.40, P = 0.34). This result is consistent with the findings of the multicenter study conducted by Daichi et al. (10) Theoretically, the first stage of DCA avoids both anastomosis and stoma creation, which should reduce operative duration. However, successful transanal exteriorization requires adequate mobilization of the proximal colon, often necessitating splenic flexure mobilization to ensure sufficient bowel length. This additional dissection may offset potential time savings, thereby narrowing the difference in operative duration between the two approaches.

Notably, DCA demonstrated a clear advantage in reducing postoperative complications. First, the risk of anastomotic leakage was significantly lower in the DCA group, with the ICA group exhibiting a 2.79-fold higher risk (OR = 2.79, 95% CI: 1.71–4.57, P < 0.0001). This finding aligns with the subgroup analysis by Pompeu et al. (1), who reported a protective effect of staged reconstruction (OR = 0.55, P = 0.04). The physiological rationale lies in the formation of firm fibrous adhesions between the serosal surface of the exteriorized colon and the anal sphincter during the initial stage. These adhesions create a tension-free environment for the definitive anastomosis and may promote neovascularization, enhancing local perfusion and tissue healing (26, 27). Second, the overall complication rate was significantly reduced in the DCA group (OR = 0.57, 95% CI: 0.41–0.79, P = 0.002), reflecting a dual benefit: reduction in major anastomotic complications and elimination of stoma-related morbidities. Previous studies have reported stoma-related complication rates ranging from 35.87% to 74.3%, including parastomal hernia, dehydration, electrolyte disturbances, and prolapse or retraction (28–30). Although DCA carries specific risks, such as ischemia of the efferent limb (31), these complications can be effectively minimized through meticulous surgical techniques, including adequate vascular preservation, secure perianal fixation, and careful patient selection (32).

Oncological safety remains paramount in LRC surgery (33, 34). This meta-analysis found no significant differences between DCA and ICA in local recurrence (OR = 1.02, 95% CI: 0.40–2.63, P = 0.98) or distant metastasis (OR = 1.51, 95% CI: 0.89–2.54, P = 0.13), consistent with findings from Raja et al. (7). These results suggest that the two-stage nature of DCA does not compromise tumor control. The key determinant of oncological outcomes is the completeness of total mesorectal excision (TME), rather than the timing or method of bowel reconstruction (35). In DCA, the first-stage procedure already achieves complete TME, ensures adequate distal and circumferential resection margins, and performs thorough lymphadenectomy. The second stage involves only gastrointestinal restoration without disturbing the oncologic integrity of the surgical field. Furthermore, proper care of the exteriorized bowel does not increase the risk of tumor seeding. As confirmed in the recent RCT by Biondo et al. (6), both groups achieved a 0% positive margin rate. Thus, DCA represents a reconstructive strategy that enhances short-term safety without sacrificing long-term oncological efficacy. It should be emphasized that the oncological outcomes in this analysis did not include key survival endpoints such as 5-year OS and disease-free survival, primarily due to the limited reporting of these metrics across the majority of the included studies. Only two RCTs reported follow-up data of ≥5 years, yet they failed to explicitly present survival outcomes. Furthermore, considerable heterogeneity was observed in the duration of follow-up among the included studies, with several having relatively short observation periods, which may limit the ability to adequately evaluate long-term oncological safety. Future studies should employ extended follow-up periods and systematically capture data on local recurrence, distant metastasis, and survival outcomes to generate more robust and comprehensive evidence for evaluating the oncological safety of DCA.

Significant heterogeneity was observed in the continuous outcome measures, likely attributable to variations in the definitions and measurement methods across studies. The heterogeneity in operation time may reflect regional differences in surgical approaches: most European studies reported a 100% laparoscopic rate, whereas early retrospective studies from China indicated that open surgery accounted for approximately 60% of cases. Differences in the definition of “hospital stay” further contributed to heterogeneity, with three studies reporting only the duration of hospitalization for the first-stage surgery, while others included both the first and second-stage surgical admissions. Furthermore, heterogeneity exists in the timing of the second-stage DCA surgery across the included studies. Although the core surgical procedures were consistent, variations in operative timing may affect intestinal blood supply and tissue healing status, thereby potentially influencing postoperative complication rates. This represents a potential source of bias in the present analysis. Sensitivity analyses demonstrated that the pooled ORs for total complications (2.53–2.95) and anastomotic leakage (2.65–2.93) remained stable upon sequential exclusion of individual studies, indicating that this heterogeneity has minimal impact on the overall results and supporting the robustness of the conclusions.

This study has several limitations. First, the majority of the included studies were retrospective; although rigorous adjustments were applied in some instances, the inherent risk of selection bias remains. Second, follow-up durations in most existing studies were limited to 3 years, leaving oncological outcomes and anal functional recovery beyond 5 years insufficiently characterized and requiring further validation. Third, certain complications such as anastomotic stenosis and ischemia of the external colon are relatively rare, and several studies had small sample sizes, which may limit statistical power. Fourth, the absence of standardized definitions and consistent reporting of neoadjuvant treatment status across studies may introduce potential confounding bias in our meta-analysis, warranting cautious interpretation of the results. Collectively, these limitations impede the feasibility of conducting meaningful subgroup analyses by tumor stage or neoadjuvant treatment exposure, thereby hindering precise identification of the optimal patient population for DCA.

## Conclusion

In conclusion, this study demonstrates that DCA significantly reduces the risks of overall complications, anastomosis-related complications, and anastomotic leakage compared with ICA in sphincter-preserving surgery for LRC. DCA demonstrates comparable performance in key perioperative outcomes and may offer particular benefits for patients seeking minimally invasive approaches, those who cannot tolerate a stoma, or those at high risk of anastomotic failure, thus representing a viable surgical alternative. Nevertheless, it should be acknowledged that the current evidence base includes a relatively high proportion of retrospective cohort studies, which may introduce selection and confounding biases. Future RCTs are warranted to confirm the robustness of these findings.

## Data Availability

The original contributions presented in the study are included in the article/**Supplementary Material**. Further inquiries can be directed to the corresponding author/s.
