# Supplementary material for: Comparison of delayed versus immediate coloanal anastomosis in patients with low rectal cancer: a recent meta-analysis
Source: Front Oncol. 2026 Jan 26;15:1749894. doi: 10.3389/fonc.2025.1749894 (PMC12883368; doi:10.3389/fonc.2025.1749894)

**Comparison of delayed versus immediate coloanal anastomosis in patients with low rectal cancer: A recent meta-analysis**

Supplementary Table 1. PICO Framework

| **PICO Element** | **Description** | **Details / Specific Criteria** |
| --- | --- | --- |
| Population | Adult patients diagnosed with low rectal cancer undergoing sphincter-preserving surgery. | Tumor Location: Rectal cancer within 7 cm from the anal verge.  Surgery Type: Patients undergoing low anterior resection (LAR) or intersphincteric resection (ISR) with coloanal anastomosis. |
| Interventions | Immediate Coloanal Anastomosis | Procedure: A one-stage procedure. The coloanal anastomosis is performed immediately following the rectal resection during the same operation.  Note: This group may include patients with or without a defunctioning stoma. |
| Control | Delayed Coloanal Anastomosis | Procedure: A two-stage procedure. In the first stage, only the proximal colon resection margin was placed outside the anus and fixed. After 1 to 4 weeks, the second stage surgery was initiated, during which the external intestinal tube was removed and the coloanal anastomosis was completed. |
| Outcomes |  | Perioperative Outcomes: Operation time; Intraoperative blood loss; Length of hospital stay;  Postoperative Complications: Total complications; Anastomosis-related complications; Postoperative anastomotic leakage;  Oncological Outcomes: Local recurrence; Postoperative distant metastasis; |
| Study design |  | Randomized controlled trial and Non-randomized controlled trial |

Supplemental Table S2. Search Strategy (Updated Search on 30st October 2025)

Database: PubMed

| Search no. | Indexed and keywords terms |
| --- | --- |
| #1 | (Rectal Neoplasms) OR (Rectal Cancer) OR (Cancer of Rectum) OR (Rectal Carcinoma) OR (Neoplasms of Rectum) |
| #2 | (Low) OR (Lower) OR (Inferior) OR (Basal) OR (Bottom) |
| #3 | (Pull - through Coloanal Anastomosis) OR (Pull through Coloanal Anastomosis) OR (Coloanal Pull - through Anastomosis) OR (Anastomosis, Pull - through Coloanal) OR (Coloanal Anastomosis, Pull - through) |
| #4 | (Turnbull - Cutait Anastomosis) OR (Turnbull Cutait Anastomosis) OR (Anastomosis, Turnbull - Cutait) OR (Turnbull - Cutaits Anastomosis) OR (DCA - related Turnbull - Cutait Anastomosis) |
| #5 | (Delayed Coloanal Anastomosis) OR (Coloanal Anastomosis, Delayed) OR (Anastomosis, Delayed Coloanal) OR (Delayed Colo - anal Anastomosis) OR (Colo - anal Anastomosis, Delayed) |
| #6 | #1 AND #2 AND #3 AND #4 AND #5 |

Database: Embase

| Search no. | Indexed and keywords terms |
| --- | --- |
| #1 | 'Low rectal cancer' OR 'Rectal cancer, low' OR 'Inferior rectal cancer' OR 'Distal rectal cancer' OR 'Low-lying rectal cancer' OR 'Rectal cancer at lower position' /exp |
| #2 | 'Pull - through coloanal anastomosis' OR 'Coloanal anastomosis by pull - through technique' OR 'Pull - through type coloanal anastomosis' OR 'Coloanal pull - through anastomosis' OR 'Anastomosis of colon and anus by pull - through' OR 'Pull - through approach for coloanal anastomosis' |
| #3 | 'Turnbull - Cutait anastomosis' OR 'DCA - related anastomosis' OR 'Turnbull - Cutait type anastomosis' OR 'Anastomosis similar to Turnbull - Cutait' OR 'Turnbull - Cutait surgical anastomosis' OR 'Modified Turnbull - Cutait anastomosis' |
| #4 | 'Delayed coloanal anastomosis' OR 'Coloanal anastomosis with delay' OR 'Anastomosis of colon and anus after delay' OR 'Post - delay coloanal anastomosis' OR 'Delayed type coloanal anastomosis' OR 'Coloanal anastomosis with postponed time' |
| #5 | #1 AND #2 AND #3 AND #4 |

Database: Web of Science

| Search no. | Indexed and keywords terms |
| --- | --- |
| #1 | TS=((Low rectal cancer) OR (Rectal cancer low - lying) OR (Low - lying rectal carcinoma) OR (Rectal carcinoma low position) OR (Low - position rectal cancer) OR (Inferior rectal cancer)) |
| #2 | TS=((Pull - through coloanal anastomosis) OR (Coloanal pull - through anastomosis) OR (Pull - through anastomosis of colon and anus) OR (Colon - anus pull - through anastomosis) OR (Anastomosis of colon to anus by pull - through method) OR (Pull - through technique for coloanal anastomosis)) |
| #3 | TS=((Turnbull - Cutait anastomosis) OR (DCA - related Turnbull - Cutait anastomosis) OR (Turnbull - Cutait coloanal anastomosis) OR (Turnbull - Cutait surgical anastomosis) OR (Anastomosis of Turnbull - Cutait type) OR (Turnbull - Cutait intestinal anastomosis)) |
| #4 | TS=((Delayed coloanal anastomosis) OR (Coloanal anastomosis delayed) OR (Delayed anastomosis between colon and anus) OR (Anastomosis of colon to anus with delay) OR (Colon - anus anastomosis postponed) OR (Postponed coloanal anastomosis)) |
| #5 | #1 AND #2 AND #3 AND #4 |

Database: cochrane library (CENTRAL)

| Search no. | Indexed and keywords terms |
| --- | --- |
| #1 | MeSH descriptor: [Rectal Neoplasms] explode all trees |
| #2 | (Rectal Cancer OR Rectum Cancer OR Carcinoma of Rectum OR Rectal Carcinomata OR Rectal Malignancy):ti,ab,kw |
| #3 | #1 or #2 |
| #4 | (Low Rectal Cancer OR “Low - lying Rectal Cancer” OR “Inferior Rectal Cancer” OR “Distal Rectal Cancer” OR “Lower Rectal Malignancy”):ti,ab,kw |
| #5 | #3 AND #4 |
| #6 | (Pull - through Coloanal Anastomosis OR “Coloanal Pull - through Anastomosis” OR “Pull - through Anastomosis of Colon and Anal Canal” OR “Colon - anal Pull - through Connection” OR “Pull - through Colo - anal Joining”):ti,ab,kw |
| #7 | (Turnbull - Cutait Anastomosis OR “DCA - related Turnbull - Cutait Anastomosis” OR “Turnbull - Cutait Coloanal Anastomosis” OR “Modified Turnbull - Cutait Anastomosis” OR “Turnbull - Cutait Type Anastomosis”):ti,ab,kw |
| #8 | #5 AND #6 AND #7 |

Database: MEDLINE

| Search no. | Indexed and keywords terms |
| --- | --- |
| #1 | (Rectal Neoplasms[MeSH Terms:noexp]) OR (Rectal Cancer[tiab]) OR (Cancer of Rectum[tiab]) OR (Rectal Carcinoma[tiab]) OR (Neoplasms of Rectum[tiab]) |
| #2 | (Low[tiab]) OR (Lower[tiab]) OR (Inferior[tiab]) OR (Basal[tiab]) OR (Bottom[tiab]) |
| #3 | (Pull-through Coloanal Anastomosis[tiab]) OR (Coloanal Pull-through Anastomosis[tiab]) OR (Anastomosis, Pull-through Coloanal[tiab]) OR (Coloanal Anastomosis, Pull-through[tiab]) |
| #4 | (Turnbull-Cutait Anastomosis[tiab]) OR (Anastomosis, Turnbull-Cutait[tiab]) OR (Turnbull-Cutaits Anastomosis[tiab]) OR (DCA-related Turnbull-Cutait Anastomosis[tiab]) |
| #5 | (Delayed Coloanal Anastomosis[tiab]) OR (Coloanal Anastomosis, Delayed[tiab]) OR (Anastomosis, Delayed Coloanal[tiab]) OR (Delayed Colo-anal Anastomosis[tiab]) OR (Colo-anal Anastomosis, Delayed[tiab]) |
| #6 | #1 AND #2 AND #3 AND #4 AND #5# |
| #7 | #6 LIMIT to English[lang] AND Humans[mesh] |

Database: China Biomedical Literature Database

| Search no. | Indexed and keywords terms |
| --- | --- |
| #1 | 直肠肿瘤 [主题词] OR 直肠癌 OR 直肠癌症 OR 直肠 carcinoma OR 直肠肿瘤（自由词） |
| #2 | 低位 OR 下部 OR 下方 OR 基底 OR 底部 |
| #3 | 拖出式结肛吻合术 OR 结肛拖出式吻合术 OR 拖出式结 - 肛吻合术 OR 结 - 肛拖出式吻合术 |
| #4 | Turnbull-Cutait 吻合术 OR Turnbull Cutait 吻合术 OR 坦布尔 - 库泰吻合术 |
| #5 | 延迟结肛吻合术 OR 结肛延迟吻合术 OR 延迟结 - 肛吻合术 OR 结 - 肛延迟吻合术 |
| #6 | #1 AND #2 AND #3 AND #4 AND #5# |

| Table S3 Comparison table of surgical approach characteristics included in the study | | | | | |
| --- | --- | --- | --- | --- | --- |
| **Study Name** | **Group** | **Surgical Approach** | **Anastomosis Method** | **Stoma Status** | **Adjunctive Techniques/Special Procedures** |
| Xiong 2016 | ICA | Midline abdominal incision + Open surgery | Hand-sewn (interrupted absorbable sutures) | Prophylactic ileostomy, stoma closure at 3 months post-op | Complete splenic flexure mobilization, pelvic drain placement, anal dilation training (6 months post-op) |
|  | DCA | Midline abdominal incision + Open surgery | Two-stage hand-sewn (second stage: 1 month post-op) | No stoma | Complete splenic flexure mobilization, colonic pull-through and fixation (≥15 cm above pubic symphysis + 3 cm below anal verge) |
| Zhou 2017 | ICA | Laparoscopy + Abdominal auxiliary incision (4~5 cm) | Stapler-assisted primary colo-rectal end-to-end anastomosis | No prophylactic stoma | TME principle, specimen extraction via auxiliary incision |
|  | DCA | Laparoscopic five-port + Transanal pull-through | Two-stage resection of externalized intestine (2 weeks postoperatively) | No prophylactic stoma | TME principle, transanal specimen extraction |
| Liu 2020 | ICA | Laparoscopy + Abdominal auxiliary incision (4~5 cm) | Double stapler-assisted primary colo-rectal anastomosis | No prophylactic stoma | TME principle, specimen extraction via auxiliary incision |
|  | DCA | Laparoscopic five-port + Transanal pull-through | Two-stage resection of externalized intestine (2 weeks postoperatively) | No prophylactic stoma | TME principle, transanal specimen extraction |
| Luo 2020 | ICA | taTME + Transanal | Manual/stapler-assisted primary colo-anal anastomosis | Routine terminal ileum protective stoma (reversal 6 months postoperatively) | taTME principle, transanal operating platform |
|  | DCA | taTME + Transanal pull-through | Two-stage colo-anal suture (2~3 weeks postoperatively) | Routine terminal ileum protective stoma (reversal 6 months postoperatively) | taTME principle, pediatric anesthesia tube support |
| Biondo 2020 | ICA | Laparoscopic (82.6%) or Open (17.4%) | Parks-type hand-sewn (interrupted sutures) | Prophylactic ileostomy, stoma closure at 6-8 months post-op | Mechanical bowel preparation + oral antibiotics, splenic flexure mobilization, nerve-sparing |
|  | DCA (Turnbull-Cutait) | Laparoscopic (91.3%) or Open (8.7%) | Two-stage hand-sewn (second stage: 6-10 days post-op) | No stoma | Mechanical bowel preparation + oral antibiotics, splenic flexure mobilization, colonic pull-through and fixation (6-7 cm), daily colonic stump irrigation check |
| Guner 2021 | ICA (CAA + Ileostomy) | Abdominoperineal approach + Open surgery | Hand-sewn (12 interrupted sutures) | Prophylactic ileostomy (right lower quadrant) | Lone-Star retractor, monopolar diathermy, complete splenic flexure mobilization, high vascular ligation |
|  | DCA (Turnbull-Cutait) | Abdominoperineal approach + Open surgery | Two-stage hand-sewn (second stage: 5-7 days post-op) | No stoma | Lone-Star retractor, monopolar diathermy, complete splenic flexure mobilization, pelvic drain placement, total parenteral nutrition (2000 kcal/day) |
| Tang 2021 | ICA | Laparoscopy + Transanal (NOSES procedure) | No intra-abdominal anastomosis; 0.5 cm intestinal externalization with natural retraction and shaping | No prophylactic stoma | Total Mesorectal Excision (TME), transanal specimen extraction |
|  | DCA | Laparoscopy + Transanal | Two-stage anoplasty (resection of externalized intestine 2~4 weeks postoperatively) | No prophylactic stoma | TME principle, transanal specimen extraction |
| Li 2021 | ICA | Laparoscopy alone | Stapler-assisted primary colo-rectal anastomosis | Prophylactic stoma required | TME principle |
|  | DCA | Laparoscopic-transanal dual-endoscopic combined | Two-stage resection of externalized intestine (after 2-week healing) | No prophylactic stoma | TME principle, transanal operating platform |
| Madbouly 2022 | ICA (TaTME-IA) | Transanal total mesorectal excision (TaTME) + Laparoscopic assistance | Stapled anastomosis | Prophylactic ileostomy, stoma closure rate at 2 years: 80% | Modified lithotomy position, ciprofloxacin prophylactic antibiotics, surgical field drainage |
|  | DCA (TaTME-TC) | Transanal total mesorectal excision (TaTME) + Laparoscopic assistance | Two-stage hand-sewn (second stage: 1 week post-op) | No stoma | Modified lithotomy position, ciprofloxacin prophylactic antibiotics, colonic pull-through and fixation to perianal skin (4 seromuscular sutures) |
| Majbar 2022 | ICA (CAA) | Open or Minimally invasive (Laparoscopy ± TaTME) | Hand-sewn side-to-end (interrupted sutures) | Prophylactic ileostomy, stoma closure at 6-8 weeks (confirmed leak-free by CT angiography), 90-day stoma rate: 84.7% | Mechanical bowel preparation + antibiotic prophylaxis, complete splenic flexure mobilization, pelvic drainage |
|  | DCA | Open or Minimally invasive (Laparoscopy ± TaTME) | Two-stage hand-sewn (second stage: 7 days post-op) | No stoma, 90-day stoma rate: 10.5% | Mechanical bowel preparation + antibiotic prophylaxis, complete splenic flexure mobilization, tension-free colonic pull-through and fixation (7-8 cm) |
| Melka 2022 | ICA | Laparoscopic approach | Hand-sewn side-to-end (interrupted sutures) | Prophylactic ileostomy, stoma closure at 6-8 weeks (confirmed leak-free by CT angiography) | Complete splenic flexure mobilization, high vascular ligation, nerve-sparing, partial/subtotal intersphincteric resection |
|  | DCA | Laparoscopic approach | Two-stage hand-sewn end-to-end (second stage: 7-10 days post-op) | No stoma | Complete splenic flexure mobilization, high vascular ligation, nerve-sparing, tension-free colonic pull-through and fixation, daily colonic stump viability check |
| Fu 2022 | ICA | Transanal Total Mesorectal Excision (taTME) | Primary colo-anal anastomosis | Prophylactic ileostomy in 5/44 cases (11.4%) | taTME principle, transanal operating platform |
|  | DCA | taTME + Transanal pull-through | Two-stage colo-anal anastomosis (2~4 weeks postoperatively) | No prophylactic stoma | taTME principle, StarPort single-port platform, intestinal support tube |
| Zhou 2022 | ICA | Laparoscopic Intersphincteric Resection (Lap-ISR) | Primary colo-anal anastomosis | Routine prophylactic ileostomy | ISR principle, laparoscopic intersphincteric dissection |
|  | DCA | Lap-ISR + Transanal pull-through | Two-stage resection of externalized intestine | No prophylactic stoma | ISR principle, transanal pull-through fixation |
| Seow-En 2024 | ICA (LTME-ICA/RTME-ICA) | Laparoscopic (LTME) or Robot-assisted laparoscopic (RTME) | Stapled anastomosis | Prophylactic ileostomy, mean stoma closure time: 5-7 months, stoma-related complication rate: 11% | Transanal minimally invasive surgery (TAMIS) platform, mechanical bowel preparation |
|  | DCA (TaTME-DCAA) | Transanal total mesorectal excision (TaTME) + Laparoscopic assistance | Two-stage hand-sewn (second stage: 7 days post-op) | No stoma | Transanal minimally invasive surgery (TAMIS) platform, mechanical bowel preparation, total parenteral nutrition support (mean 5 days) |
| Daichi 2025 | ICA | Laparoscopic (70%), Robot-assisted (30%) or TaTME (61%) | Hand-sewn | Prophylactic ileostomy, permanent stoma rate at 1 year: 16% | Lloyd-Davis position, splenic flexure mobilization, intersphincteric resection (84%) |
|  | DCA | Laparoscopic (70%), Robot-assisted (30%) or TaTME (54%) | Two-stage hand-sewn full-thickness (second stage: 7 days post-op) | No stoma, permanent stoma rate at 1 year: 1% | Lloyd-Davis position, splenic flexure mobilization, Bianco technique (colonic stump fixed to rectal cuff upper edge) |

Note: ICA = Immediate coloanal anastomosis; DCA = Delayed coloanal anastomosis; TME = Total mesorectal excision; TaTME = Transanal total mesorectal excision; LTME = Laparoscopic total mesorectal excision; RTME = Robot-assisted total mesorectal excision; CAA = Coloanal anastomosis; CT = Computed tomography; SD = Standard deviation.

| Table S4 Risk of bias for the included studies, based on the ROBINS-I tool | | | | | | | | | |
| --- | --- | --- | --- | --- | --- | --- | --- | --- | --- |
| Author | Year | Type of bias | | | | | | | Overall rating |
|  |  | Confounding | Selection of participants | Exposure assessment | Misclassification during follow-up | Missing data | Measurement of the outcome | Selective reporting of the results |  |
| Xiong | 2016 | Low | Moderate | Low | Low | Low | Moderate | Low | Moderate |
| Zhou | 2017 | Moderate | Moderate | Low | Low | Low | Moderate | Low | Moderate |
| Liu | 2020 | Moderate | Moderate | Low | Low | Low | Moderate | Low | Moderate |
| Luo | 2020 | Low | Low | Low | Low | Low | Low | Low | Low |
| Guo | 2021 | Low | Moderate | Low | Low | Low | Low | Low | Moderate |
| Tang | 2021 | Low | Moderate | Low | Low | Low | Low | Low | Moderate |
| Li | 2021 | Moderate | Low | Low | Low | Low | Moderate | Low | Moderate |
| Guner | 2021 | Moderate | Moderate | Low | Low | Low | Low | Low | Moderate |
| Madbouly | 2022 | Low | Low | Low | Low | Low | Low | Low | Low |
| Majbar | 2022 | Moderate | Moderate | Low | Low | Low | Low | Low | Moderate |
| Melka | 2022 | Low | Moderate | Low | Low | Low | Low | Low | Moderate |
| Fu | 2022 | Low | Moderate | Low | Low | Low | Low | Low | Moderate |
| Seow-En | 2024 | Low | Moderate | Low | Low | Low | Low | Low | Moderate |
| Daichi | 2025 | Moderate | Low | Low | Low | Low | Low | Low | Moderate |
| ROBINS-I: risk of bias in non-randomized studies of interventions; NA=not applicable | | | | | | | | | |

Supplementary Figure 1. Risk of bias assessment using the Cochrane Risk of Bias Tool version 2.


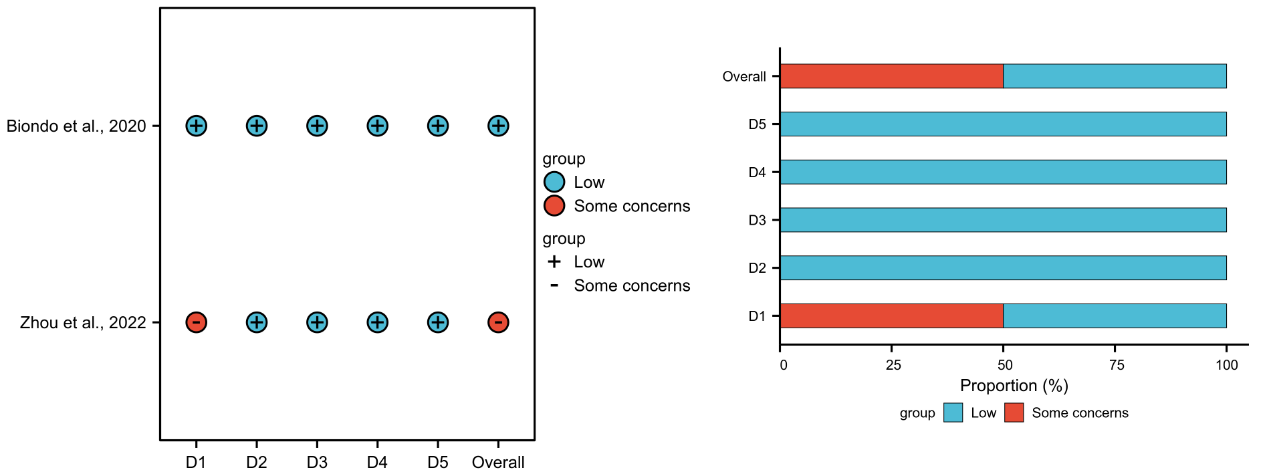


Supplementary Figure 2. Funnel plot and Egger test results. (A) Operative time; (B) Intraoperative blood loss; (C)Length of stay in hospital；(D) Overall complication; (E) Anastomotic-related complications; (F) Anastomotic leak; (G) Local recurrence; (H) Distant metastases.


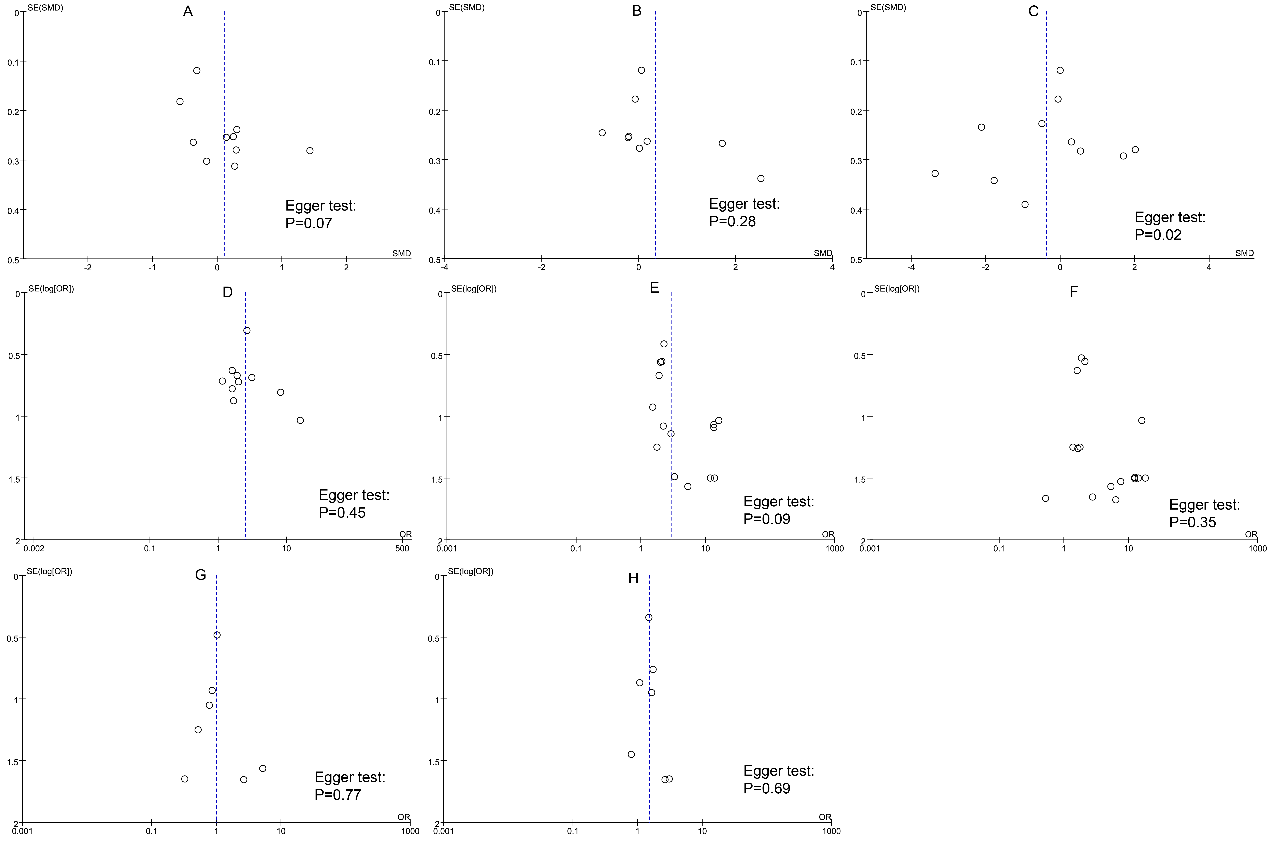

Supplement: Supplementary file 1 [file Table1.docx]
